# Parenting through place‐of‐care disruptions: A qualitative study of parents' experiences of neonatal care

**DOI:** 10.1111/hex.13933

**Published:** 2023-12-18

**Authors:** Caroline Cupit, Alexis Paton, Elaine Boyle, Thillagavathie Pillay, Josie Anderson, Natalie Armstrong, the OPTI‐PREM team

**Affiliations:** ^1^ Department of Population Health Sciences University of Leicester Leicester UK; ^2^ Centre for Health and Society Aston University Birmingham UK; ^3^ Research Institute for Health Related Sciences University of Wolverhampton Wolverhampton UK; ^4^ BLISS London UK

**Keywords:** community of care, management, neonatal care, parent involvement, parenting, policy, qualitative methods

## Abstract

**Introduction:**

Neonatal care is complex, involving multiple people and technologies within a community of care. When preterm babies are cared for far from home and/or transferred between units, the whole community of care (and particularly parent participation) is disrupted. Although previous studies have captured subjective experiences of parents, there has been little research exploring the material practices undertaken by parents as a consequence of place‐of‐care decisions, or the social organisation of those practices.

**Methods:**

As part of a wider study exploring optimal place‐of‐care, semistructured interviews were conducted between July 2018 and October 2019 with 48 parents (36 families) with one or more preterm babies (born at 27–31 weeks gestation) cared for in a neonatal unit in the last 12 months.

**Findings:**

We highlight parents' labour‐intensive and stressful work to: (1) parent in the neonatal care community (an oversight role that goes beyond contemporary notions of ‘involvement’); (2) create continuity amid place‐of‐care disruptions; and (3) adapt to the managerial logics of neonatal care settings. Our analysis focuses on the work generated by managerial systems that organise place‐of‐care decision‐making and other efficiency‐focused practices. Parents are absorbed into negotiating institutional systems and diverted from routine parenting activities.

**Conclusion:**

Those involved in the organisation and management of neonatal care should take account of how managerial systems impact parents' workload, ability to participate in their baby's community of care and, ultimately, on the wellbeing and development of babies and their families.

**Patient or Public Contribution:**

The OPTI‐PREM study embedded parents' experiences of neonatal care into the research, through a discrete workstream that employed qualitative methodology to capture parents' experiences—as reported in this paper. The OPTI‐PREM project was also supported by a Bliss volunteer parent panel, which was involved in designing and overseeing the research. Bliss ‘champion[s] the right for every baby born premature or sick to receive the best care by supporting families, campaigning for change and supporting professionals and enabling life‐changing research’ (https://www.bliss.org.uk/about-us/about-bliss). A representative of Bliss is a co‐author of this manuscript, and a parent representative (named in the Acknowledgements) provided feedback during its preparation.

## 1 INTRODUCTION

Neonatal units are sites in which communities of people provide care for preterm babies and those born full term but unwell.^1^ These communities include a variety of professional staff, parents and volunteers who come and go from the unit, work with an array of medical technologies (e.g., machines, medicines) and have to constantly negotiate their caring responsibilities in relation to others. Parents are integral to a baby's community of care. Whether paid or unpaid, care is labour‐intensive and is multiple, diverse and shifting as it intersects with the work of others. Within a neonatal unit, there are multiple communities of care that overlap and intersect as staff (and various technologies) are shared between them.

In this paper, we consider what parents have to do to participate in their baby's community of care. We use Smith's conceptualisation of ‘work’ (‘anything that people do that takes time, effort and intent’)^2^ to particularly focus on how parents create continuity when care is disrupted by transfers between units. Our analysis follows a previous report from this study, in which we showed how networks of neonatal units are organised around matching the (anticipated or known) clinical needs of the neonatal baby population (demand) with the availability of expertise and technologies (capacity).^3^ This demand and capacity management across a regional geography necessitates that preterm babies are cared for in more or less intensive care facilities according to their individual clinical needs and in relation to the needs of other babies and the resources available. Systems of demand and capacity management (which include contractual funding arrangements, safe staffing protocols, etc.) tightly coordinate the work of staff, who are consequently drawn into ongoing and labour‐intensive work to manage their resources—‘juggling’ babies between units to free‐up cots, staffing and medical technologies for others deemed in higher need. The clinical needs of the baby are of fundamental importance within such systems, but *how practically* these various needs are understood and managed is determined by the dominant managerial logics we have outlined. Other considerations (e.g., babies' needs for parents to be physically and emotionally present) have less traction within these management systems.

This paper follows directly from our previous analysis of place‐of‐care decision‐making in neonatal units.^3^ We highlight how, as staff work is orientated towards the management of demand and capacity, there are knock‐on effects on the whole community of care. In particular, we show how parents' energies are directed into creating continuity for their baby. This impacts their ability to undertake more usual parenting activities,and may have consequences for babies' development, as well as parents' wellbeing and subjective experiences.

### 1.1 Social organisation of parents' participation

Communities of care, such as those in neonatal units, are socially organised—shaped by various policies and protocols^4^ as well as wider structures of ‘place and space’.^1^ Such systems of governance organise care within and beyond a particular unit,^3^, ^4^ and structure the context for parental participation. The importance of parental (especially maternal) participation in neonatal care is well documented.^5^ Particularly important to parents is knowledge and control over their baby's care, the removal of which is associated with significant trauma.^6^, ^7^, ^8^, ^9^ Following delivery, parents need support to (re)establish oversight and control^10^, ^11^, ^12^, ^13^—a role that has to be negotiated within the baby's emerging community of care. Good‐quality information, communication and positive relationships between parents and neonatal staff are central to establishing parental participation that is manageable for the individual family,^14^, ^15^, ^16^, ^17^ enabling parents to ‘hope’ even in the face of severe illness, disability or death of their baby^18^ and to manage the stress of parenting a preterm baby.^19^, ^20^

In recognition of the importance of parental participation, models such as ‘family integrated care’ (FICare)^21^, ^22^, ^23^ (founded on a conceptual model of ‘family‐centred care’^24^) are increasingly embraced by service providers. In the United Kingdom, Bliss, the charity for babies born premature or sick, introduced a ‘Baby Charter’ to champion family‐ centred care.^25^ The British Association for Perinatal Medicine has also recently developed a framework for practice, actively supporting FICare for neonatal services in the United Kingdom.^26^ Such policies recognise multiple dimensions of family integration.^25^ Evaluations of various family‐centred care initiatives suggest that encouraging family participation may have a positive impact on babies' clinical outcomes.^22^ However, the value of these formalised models, and the extent to which parents should be *expected* to be involved in care, is still debatable.^15^, ^27^, ^28^, ^29^

Naylor et al.^1^ conceptualise parental participation as part of a community of care, highlighting key activities in which parents participate (e.g., touch, monitoring, feeding, hygiene, communication with staff, child advocacy)—and illustrating how intricately parental work is bound up with the work of others. Whilst arguing that care is ‘co‐produced’, they nevertheless expose the multiple different priorities that may arise within a community of care and tensions that are ‘negotiated by actors with differing levels of power’. Similarly, Navne and Svendson's^30^ study of staff experiences of decision‐making highlights tensions between medical authority and parental participation, which they show does not always fit neatly into ‘already established routines and knowledge spheres’.

Socially organised systems of knowledge and practice shape how parents can participate in their baby's community of care.^1^, ^4^, ^29^ Such systems are based on economic modelling and network structures that centralise specialist care in a small number of urban locations. High‐intensity units are much more costly to run and have a limited capacity,^31^, ^32^, ^33^, ^34^, ^35^, ^36^ necessitating systems of demand and capacity management through which babies are ‘juggled’ across differently resourced units in a network—as we have highlighted in a previous paper.^3^ Clinical staff are ultimately responsible for making transfer decisions, choreographing that care^30^ and ‘juggling’ babies in accordance with management priorities.^3^ Although communication between staff and parents goes a long way to helping parents feel more comfortable with transfers,^10^, ^37^, ^38^ parents are largely excluded from the choreography of neonatal care^38^, ^39^, ^40^ as it is a function directly related to the efficient management of neonatal units and networks.^41^

This paper draws particular attention to what Aagaard et al.^42^ describe as a ‘disruption of parenthood’ that is produced by a distant place of care and/or transfer of care location. Aagaard et al.'s meta‐study investigated parents' experiences of neonatal transfer and the impact these experiences have on parenthood. They argue that becoming a parent is a journey that requires sufficient time to adjust to the existential changes that parenthood brings and develop the skills to parent. As such, neonatal transfer disrupts this process by interfering with a parent's ability to be close to their infant, which can then threaten their own identity as a parent. This interruption can have severe consequences, with parents of transferred infants reporting feeling ‘confused, disappointed, left behind, robbed or useless’. Their study lends support to policy ambitions that position parents ‘at the centre of their baby's care’,^25^ and challenges iterations of family‐centred care that fail to offer meaningful support for parenthood, as defined in broad experiential terms.

Our study builds on Aagaard et al.'s^42^ analysis by focusing attention on *situated and material parenting work* in neonatal care communities (rather than on existential notions of parenthood)—and the *social organisation* of those practices. Our aim is to show parenting work as constricted by managerial logics—systems that not only take account of the clinical needs of the baby, but are designed to maximise *efficient use of resources* across a neonatal network. Specifically, we contribute to discussions of parental participation in neonatal care by highlighting the effort and resources that parents have to mobilise to establish and maintain a central role in their baby's care amid place‐of‐care disruptions. Many parents' energies are so comprehensively diverted into creating continuity for their neonate that more nurturing and practical aspects of parenting (including for other children) are significantly restricted.

## 2 METHODS

This paper reports on data collected as part of the OPTI‐PREM study^43^—a mixed‐methods study to explore the best place of care for babies born 27–31 weeks gestation. (In the absence of clear evidence to guide optimal place of care, babies born at 27–31 weeks are currently born and cared for across both neonatal intensive care unit [NICU] and local neonatal unit [LNU].) Our ethnographic component of the study was carried out in two Neonatal Operational Delivery Networks in England. Within each network, a NICU and two attached LNUs were included (six neonatal units in total). From January to October 2018, observations were conducted to explore decision‐making about place of care in a real‐world context. Interviews were also carried out with staff and parents. In a previous paper (based on observations and *staff* interviews), we reported on how place‐of‐care decisions are made in real‐world neonatal practice and pointed to the managerial systems organising these decisions.^3^ In this paper, we draw on further interviews with *parents* to explore how managerial systems impact on their participation in the care of their baby.

### 2.1 Recruitment

#### 2.1.1 Parents were recruited to interview via two routes

First, parents were made aware of the study by healthcare professionals at the sites where observations were taking place. All eligible parents (i.e., those with a baby born between 27 and 31 weeks) who had a baby in the units during periods of observation were approached for an interview by the researcher or clinical team overseeing the care of the baby. These real‐time interviews (i.e., undertaken with parents concurrently caring for babies on a neonatal unit) took place on the units in a quiet side room.

Second, we also undertook retrospective interviews with parents who had recent (but not contemporaneous) experience of neonatal care. Most parents were approached through the charity, Bliss,^44^ which utilised their social media channels to recruit parents with experience of neonatal care in the previous year. A few were recruited through their contact with a researcher in a neonatal unit. After the first general appeal, we found that responses were primarily from parents motivated to provide positive feedback about the care provided to their baby, and they benefited from a relatively high socioeconomic status (as understood by the researchers based on interview discussions). Concerned that the experiences of this group might be divergent from a ‘typical’ experience (including some of those observed during fieldwork), we subsequently sent out a second appeal specifically seeking parents who had found the experience ‘particularly challenging for practical reasons (e.g., finances, family, culture/religion, English language skills, employment), in addition to their concerns about their baby's medical condition and care’. Our rationale was to encourage responses from a more diverse group of parents than are often represented in research studies. As a result, several more complex accounts of neonatal care were recorded and these strengthened the findings of this study.

### 2.2 Data collection and analysis

Data were collected and analysed by A. P. and C. C. (experienced social scientists). Written consent for interviews was obtained. Thirty‐six interviews were conducted (14 real time; 22 retrospective). These included 48 parents (12 of the interviews were with both mother and father). Interviews lasted up to 1 h and were audio‐recorded. Some were conducted face to face and others over the telephone. A topic guide developed through literature review and discussions within the project team (including parent representatives) was used, but conversations were also guided by the issues raised by participants. Interviews were transcribed verbatim and anonymised. Analysis was inductive and interpretative, with A. P. using the constant comparative method to undertake initial coding.^45^ C. C. then drew on an analytic approach known as ‘institutional ethnography’^46^, ^47^, ^48^, ^49^ to particularly consider the ‘work’ involved for parents (referring to a wide range of activities that take time and effort).^50^ NVivo software was used to organise and retrieve data.

This work was approved by North East Tyne and Wear South REC (IRAS 212304).

## 3 FINDINGS

We present our findings under three headings: (1) parenting in the neonatal care community; (2) creating continuity amid place‐of‐care disruptions; and (3) adapting to the managerial logics of neonatal care.

### 3.1 Parenting in the neonatal care community

Parenting in neonatal care has been shown previously to involve touching, monitoring, feeding and hygiene‐related tasks.^1^ In this study, we found that an important aspect of parenting a preterm baby was also overseeing the care community—taking ‘responsibility for their infant's care’^42^ whilst also recognising their need for the wider community of technologies and expertise in and beyond the neonatal unit. Many parents praised the staff who they watched at work:

I was there all day, twenty four seven really so I just got to know all the staff. […] they're just brilliant aren't they? I just couldn't fault them in any way. (Mother16)

As part of overseeing care, parents valued good communication from staff:

We knew from the very beginning what the process was going to be, and they kept us in the loop, they always told us what's coming next. (Father72)

Conversely, it was difficult for parents when staff did not have the answers, and they became concerned about safety if this was apparently jeopardised by a lack of continuity (e.g., knowledge handover):

The nurse wrote [on the notes] ‘[Baby] had a really good night, but her heart rate dropped around two am’ and they'd say, ‘Have you had a chance to look through?’ And I'd say ‘Yeah, what happened at two o'clock?’ and she was like, ‘I don't know’. (Mother77)

From a parental perspective, the quality of care, regardless of whether the baby was transferred, related to parents' perceptions of staff skill and attentiveness, and whether (or not) these could adequately substitute for that of parents when they were absent from the cot‐side.

I wanted to see, I didn't know the nurses there and I wanted to see how they were. And then after those first two nights I was like ‘okay, I'm happy now to leave her because now I know’. (Mother64)

Consistency of nursing and medical oversight was important, especially for parents who had limited capacity to be physically present to oversee care and/or were under other pressures:

[My time in the neonatal unit was] a bit all over the place, like, my daughter was only fourteen months, so my boyfriend quit his job to help with childcare and visits. And we would go in the day time for about an hour or two hours, sometimes with our daughter, sometimes without. And then I would spend the majority of my day with my daughter at home, and I'd get her to bed about seven o'clock in the evening, then I'd go straight back to hospital until three or four in the morning. I'd come back for a few hours to sleep and then get up with my daughter again, so it was very all over the place. I felt torn between the two. […] (Mother78)

Building relationships within a consistent community of care freed parents to engage in more natural parenting, including, for example, touching, monitoring, feeding and hygiene^1^:

We saw probably two consultants and it was consistent. So it wasn't somebody different every day, and you soon get used to learning what they're doing with the hourly obs and stuff. That was explained and you could just sit with them, and you can help them, and they encourage you to get involved. (Mother23)

### 3.2 Continuity work to minimise place‐of‐care disruptions

Previously, we showed how transfers are often enacted in response to managerial concerns about demand and capacity.^3^ Place‐of‐care disruptions disrupt parenting work, limiting parents' ability to be there to oversee care and draining their time, emotional and financial resources:

We couldn't really stay as often as we'd have liked at [distant hospital], but we did try as much as we could. […] You can't just say, ‘Well I'm just going to go to see the baby for a couple of hours’, because there's that much you have to sort out just to go to see the baby, you know, feeding the kids, travelling, money costs as well. […] (Mother36)

Parents highlighted that moving hospitals meant ‘starting again’—(re)negotiating their role within, and their oversight of, their baby's care community. This is hard work, which strives towards continuity for the baby and is based on in‐depth knowledge of that baby:

I think for me it's the ongoing care [that's important], because you know as soon as you move hospitals you're going to have to start again. And you've worked so hard: you know the routine, you know the staff, they know [baby]. (Mother71)

A new location necessitated that parents (re)assure themselves that the baby was being well cared for:

Different hospitals have different procedures, it was like, ‘Oh, what's that?’ And she was like, ‘Oh, it's something we do for all our babies, don't worry’. And you're like, ‘But I want to worry’. Not that I [really] want to worry, but I want to know what's [pause] happening. (Mother77)

As the above excerpt highlights, parents' oversight role can feel intangible to parents and be difficult to communicate, perhaps contributing to a limited emphasis in the research literature. We see here that it is much more than just ‘worry’; it is about surveillance—*knowing what is happening*—and through surveillance creating continuity.

There are multiple practical elements to creating continuity when a baby is transferred from one site to another. As well as the stress of transfer itself, parents had to take on board a raft of new information, learning how to navigate different hospital layouts, systems and equipment:

[As part of the induction, the nurse said] ‘This is the milk kitchen, this is the linen store, this is this, this is that.’ And we were going round and I'm thinking, ‘I really just want to sit down’ [laughs]. And then it was, ‘Oh, you need to hire one of our pumps, so we need a deposit for the pump, can you get (…)’ Okay, fine sure, I'll book that out somehow, and after all this, eventually, they just let me sit next to her incubator again. (Mother75)

Parental participation was made more difficult by inconsistent systems and procedures between units. Inconsistencies spanned provision of equipment (e.g., breast pumps), accommodation and care procedures:

Everyone agrees that [there's] a lack of consistency of equipment between hospitals. […] This also means that there are often interventions to ‘correct’ between the different standards and baby can be poked and prodded three times just so that leads or drugs comply with new standards. Donor milk is not consistent across sites either. (Observation, Site 6, LNU)

A baby transferred from Site X had no records. Medical staff were aware of baby and had discussed him during handover, but on electronic system there was nothing […] (Observation, Site 1, NICU)

Such inconsistencies required that parents expend time and effort to work out what was happening (create continuity) amid the disruption caused by relocation (baby ‘juggling’). This inevitably distracted from the important activities involved in practical parenting.

### 3.3 Adapting to the managerial logics of neonatal care settings

Parents find it challenging to create continuity for their baby, especially when this has to be renegotiated within a baby's new community of care. Disruption to the community of care (and parents' roles within it) exacerbates tensions between parental knowledge (e.g., of their baby's need for their closeness and nurture) and managerial systems of knowledge:

[Hospital A] teach their parents to tube feed their prem babies whereas, when I went to [Hospital B], the sister was like ‘No, we don't do that here, the nurses will do that for you’. And I didn't want them to, I wanted to do that myself. And I wasn't allowed. And then, I wasn't allowed to hold them and I said, ‘I feel like it's just taking them away from me’. (Mother77)

The excerpt above illustrates that systems of demand and capacity management^3^ permeate into the whole operation of neonatal units—organising not only the ‘juggling’ of babies from one unit to another but also choreographing staff activities and putting their time under pressure. This means that when units make space for nurses to support parent participation in care (e.g., tube‐feeding), the time involved (to ensure this participation is undertaken in line with unit operations) may threaten the efficiency of the unit.

Parents have to fit into an operational schedule, which determines how and when parents can learn and then put their learning into practice:

There are plenty of mums that would also go to do things with their baby and then be told, ‘Oh no, we can't do that now’, I know that that wasn't just me. Some mums would cause absolute havoc in there and be like, ‘No, that's my baby, I don't care what you say. If he gets poorly, you can look after him because he's in the place to be looked after, but I'm doing it my way because that's my child’. (Mother78)

When parents are unable to adapt to the system, they ‘struggle’ because their parenting work (e.g., their ability to ensure the wellbeing of their baby) is undermined:

All [the nurse] kept saying to me was like, ‘You've just got to adapt’. And it was like, ‘I don't want to adapt’. And I struggled because I had to commute and get the train every day, leaving them on a night, and I said to the nurse, ‘Please don't let them cry, they're not used to crying’. […] (Mother77)

This need to adapt to the system, in the context of the stresses of having a very sick baby, creates an extra pressure on parents, which can be overlooked:

That's the one thing that I could honestly say throughout the whole of our neonatal journey was that I feel that the mental health support for parents is quite poor. […] Someone just to be there to say, ‘if you need to talk to anybody, this is who you can go and talk to’ because I just felt that that was something that was so lacking. (Mother65)

As this study's data show, managerial logics (about how and when parents can participate in care) create difficulties, particularly for parents with challenging social circumstances, who are unable to easily fit into units' systems and processes. Tensions are generated between parents' desire to create continuity and managerial systems of demand and capacity management. Ultimately, clinical staff (working within their unit's managerial systems) have authority^30^ and, when they are drawn into managerial work to ‘juggle’ babies from one unit, a cascade of other work is created for both staff and parents. Here, we have particularly highlighted the work of parents that includes (re)building relationships in the community of care. As the community of care is delicately constituted, disruption has a knock‐on impact on parents' ability to participate and, in the longer term, on the baby's and family's development and wellbeing.

## 4 DISCUSSION

In this paper, we have highlighted that, although parenting within the neonatal community of care does involve practical and emotional work (e.g., touching, monitoring, feeding, hygiene, etc.), it also includes overseeing a baby's care, which is of pre‐eminent importance to parents. This oversight, along with more familiar forms of parenting, is disrupted when care is delivered far from home or a baby is transferred between hospitals.^42^, ^51^ Supporting parenthood is not only crucial in improving parents' subjective experience of neonatal services but is also likely to reduce stress on the baby, improve short‐ and long‐term developmental outcomes and minimise the need for health intervention.^22^, ^52^, ^53^, ^54^

This paper follows from our previous analysis of managerial systems that organise decisions about place of care—and lead to transfers that are not always clinically necessary and very often burdensome for parents.^3^ Management systems that organise place of care can have considerable knock‐on effects, not only creating stress for parents^10^, ^37^, ^38^, ^39^, ^55^ but also precipitating new work to create continuity amid those place‐of‐care disruptions. Indeed, within the community of care, parents undertake a significant amount of the work required to maintain continuity for their babies. This form of parental participation is an invaluable resource, with a proven positive impact on health outcomes, and the potential to support efficiency and value by relieving pressure on clinical staff. However, it should not be assumed that parents can simply incorporate this continuity work as part of their more obvious parenting tasks.

Faced with exhaustion resulting from pregnancy, labour and the stress of complications, parents need help to navigate the managerial systems into which they are suddenly inserted. This might involve, for example, better standardisation of systems (e.g., for donor milk) between units; better systems for communicating processes with parents; and a greater attention to the emotional and rest needs of parents following delivery. As parents from disadvantaged backgrounds find it particularly difficult to incorporate neonatal parenting into their (often complicated) everyday lives,^56^ policymaking should specifically assess the burden placed on parents from various socioeconomic backgrounds and their consequent ability to maintain continuity work (alongside the many other elements of parental care) in the face of place‐of‐care disruptions. This is important because continuity work is likely to add to parental stress, which has been related to maladaptive parenting and to long‐term developmental problems in the child.^57^, ^58^

Our findings suggest that continuity should be more central in discussions about the best place of care—a finding supported by evidence from neonatal and other services.^59^, ^60^ Continuity has been recognised as a casualty of a modern focus on value and efficiency,^61^ which we see in policymaking design and delivery of neonatal services. Although stakeholders are rightly concerned to ensure value and efficiency (financial calculations based on clinical outcomes and costs)—and continuity should not be the *only* factor to guide policymaking—we are arguing that the burden involved in managing continuity of care should be kept in view (and mitigated) as other managerial goals are prioritised within an increasingly industrial healthcare system.^61^

## 5 CONCLUSION

Parenting a baby in neonatal care is difficult. Parents have to negotiate both their private social worlds and their participation in the (medical) neonatal care community. Neonatal care that is far from home and/or changes to place‐of‐care create significant disruption to parenting work. Whilst neonatal transfer is often inevitable due to the clinical needs of babies and the geographical patterning of resources, parenting remains central to good neonatal care—and we have highlighted that this includes oversight and continuity work alongside more mundane parenting tasks. Those involved in the organisation and management of neonatal care should take account of how managerial systems that increase baby ‘juggling’ (choreography of transfers from one site to another)^3^ are impacting on the neonatal community of care—and particularly on parenting and the wellbeing and long‐term outcomes of babies.

## THE OPTI‐PREM STUDY TEAM

Natalie Armstrong, Victor L. Banda, Vasiliki Bountziouka, Caroline Cupit, Kelvin Dawson, Elaine M. Boyle, Elizabeth S. Draper, Abdul Qader T. Ismail, Bradley Manktelow, Neena Modi, Alexis Paton, Oliver Rivero‐Arias, Sarah E. Seaton, Miaoqing Yang and Thillagavathie Pillay (Chief Investigator).

## AUTHOR CONTRIBUTIONS

*Obtained funding*: Thillagavathie Pillay, Elaine Boyle and Natalie Armstrong. *Study design*: Natalie Armstrong. *Data collection*: Alexis Paton and Caroline Cupit. *Data analysis*: Caroline Cupit. *Drafted manuscript*: Caroline Cupit. *Reviewed and commented on manuscript*: Thillagavathie Pillay, Elaine Boyle, Natalie Armstrong, Alexis Paton and Josie Anderson. *Interpreted to neonatal practice*: Thillagavathie Pillay and Elaine Boyle. *Conceptualised and critically revised the manuscript*: Caroline Cupit. All authors approved the final version.

## CONFLICT OF INTEREST STATEMENT

The authors declare no conflict of interest.

## ETHICS STATEMENT

This study involves human participants and was approved by North East Tyne and Wear South REC (IRAS 212304). Participants gave informed consent to participate in the study before taking part.

## Supporting information

Supporting information.

Supporting information.

## Data Availability

No data are available.

## References

[1] Naylor L , Clarke‐Sather A , Weber M . Troubling care in the neonatal intensive care unit. Geoforum. 2020;114:107‐116. 32565554 10.1016/j.geoforum.2020.05.015 PMC7295500

[2] Smith DE . Institutional Ethnography: A Sociology for People. Rowman & Littlefield; 2005:271.

[3] Cupit C , Paton A , Boyle E , Pillay T , Armstrong N . Managerial thinking in neonatal care: a qualitative study of place of care decision‐making for preterm babies born at 27–31 weeks gestation in England. BMJ Open. 2022;12(6):e059428. 10.1136/bmjopen-2021-059428 PMC9237905 35760541

[4] Ringham C , Rankin J , Marcellus L . The social organization of neonatal nurses' feeding work. Neonatal Netw. 2020;39(5):283‐292. 32879044 10.1891/0730-0832.39.5.283

[5] Gallegos‐Martínez J , Reyes‐Hernández J , Scochi CGS . The hospitalized preterm newborn: the significance of parents' participation in the neonatal unit. Rev Lat Am Enfermagem. 2013;21(6):1360‐1366. 24402347 10.1590/0104-1169.2970.2375

[6] Cleveland LM . Parenting in the neonatal intensive care unit. J Obstet Gynecol Neonatal Nurs. 2008;37(6):666‐691. 10.1111/j.1552-6909.2008.00288.x 19012717

[7] Spinelli M , Frigerio A , Montali L , Fasolo M , Spada MS , Mangili G . ‘I still have difficulties feeling like a mother’: the transition to motherhood of preterm infants mothers. Psychol Health. 2016;31(2):184‐204. 26359768 10.1080/08870446.2015.1088015

[8] Palmquist AEL , Holdren SM , Fair CD . “It was all taken away”: lactation, embodiment, and resistance among mothers caring for their very‐low‐birth‐weight infants in the neonatal intensive care unit. Soc Sci Med. 2020;244:112648. 31707144 10.1016/j.socscimed.2019.112648

[9] Tomori C , Gribble K , Palmquist AEL , Ververs MT , Gross MS . When separation is not the answer: breastfeeding mothers and infants affected by COVID‐19. Matern Child Nutr. 2020;16(4):13033. 10.1111/mcn.13033 PMC7267086 32458558

[10] Rowe J , Jones L . Facilitating transitions. Nursing support for parents during the transfer of preterm infants between neonatal nurseries. J Clin Nurs. 2008;17(6):782‐789. 18279281 10.1111/j.1365-2702.2007.02118.x

[11] Kearvell H , Grant J . Getting connected: how nurses can support mother/infant in the neonatal intensive care unit. Aust J Adv Nurs. 2010;27(3):75.

[12] Weis J , Zoffmann V , Egerod I . Enhancing person‐centred communication in NICU: a comparative thematic analysis. Nurs Crit Care. 2015;20(6):287‐298. 24237931 10.1111/nicc.12062

[13] Treherne SC , Feeley N , Charbonneau L , Axelin A . Parents' perspectives of closeness and separation with their preterm infants in the NICU. J Obstet Gynecol Neonatal Nurs. 2017;46(5):737‐747. 10.1016/j.jogn.2017.07.005 28802557

[14] De Rouck S , Leys M . Information needs of parents of children admitted to a neonatal intensive care unit. Patient Educ Couns. 2009;76(2):159‐173. 19321288 10.1016/j.pec.2009.01.014

[15] Wigert H , Dellenmark Blom M , Bry K . Parents' experiences of communication with neonatal intensive‐care unit staff: an interview study. BMC Pediatr. 2014;14(1):304. 25492549 10.1186/s12887-014-0304-5 PMC4276021

[16] Ballantyne M , Orava T , Bernardo S , McPherson AC , Church P , Fehlings D . Parents' early healthcare transition experiences with preterm and acutely ill infants: a scoping review. Child Care Health Dev. 2017;43(6):783‐796. 28370174 10.1111/cch.12458

[17] Adama EA , Adua E , Bayes S , Mörelius E . Support needs of parents in neonatal intensive care unit: an integrative review. J Clin Nurs. 2022;31(5‐6):532‐547. 34312923 10.1111/jocn.15972

[18] Charchuk M , Simpson C . Hope, disclosure, and control in the neonatal intensive care unit. Health Commun. 2005;17(2):191‐203. 15718196 10.1207/s15327027hc1702_5

[19] Miles MS , Funk SG , Kasper MA . The neonatal intensive care unit environment: sources of stress for parents. AACN Adv Crit Care. 1991;2(2):346‐354. 10.4037/15597768-1991-2022 2021521

[20] Al Maghaireh DF , Abdullah KL , Chan CM , Piaw CY , Al Kawafha MM . Systematic review of qualitative studies exploring parental experiences in the neonatal intensive care unit. J Clin Nurs. 2016;25(19‐20):2745‐2756. 27256250 10.1111/jocn.13259

[21] O'Brien K , Bracht M , Robson K , et al. Evaluation of the Family Integrated Care model of neonatal intensive care: a cluster randomized controlled trial in Canada and Australia. BMC Pediatr. 2015;15(1):210. 26671340 10.1186/s12887-015-0527-0 PMC4681024

[22] O'Brien K , Robson K , Bracht M , et al. Effectiveness of Family Integrated Care in neonatal intensive care units on infant and parent outcomes: a multicentre, multinational, cluster‐randomised controlled trial. Lancet Child Adolesc Health. 2018;2(4):245‐254. 30169298 10.1016/S2352-4642(18)30039-7

[23] FiCare . About FICare. Family Integrated Care; 2020. http://familyintegratedcare.com/about-ficare/

[24] Reid S , Bredemeyer S , Chiarella M . Integrative review of parents' perspectives of the nursing role in neonatal family‐centered care. J Obstet Gynecol Neonatal Nurs. 2019;48(4):408‐417. 10.1016/j.jogn.2019.05.001 31150595

[25] Bliss . Bliss Baby Charter. Bliss; 2022. https://www.bliss.org.uk/health-professionals/bliss-baby-charter/what-is-the-baby-charter/whats-involved

[26] British Association of Perinatal Medicine . Family Integrated Care. British Association of Perinatal Medicine; 2021. https://www.bapm.org/resources/ficare-framework-for-practice

[27] Jiang S , Warre R , Qiu X , O'Brien K , Lee SK . Parents as practitioners in preterm care. Early Hum Dev. 2014;90(11):781‐785. 25246323 10.1016/j.earlhumdev.2014.08.019

[28] Warre R , O'Brien K , Lee SK . Parents as the primary caregivers for their infant in the NICU: benefits and challenges. NeoReviews. 2014;15(11):e472‐e477.

[29] Ringham CL , MacKinnon K . Mothering work and perinatal transfer: an institutional ethnographic investigation. CJNR. 2021;53(1):10‐11. 10.1177/0844562119884388 31684752

[30] Navne LE , Svendsen MN . Careography: staff experiences of navigating decisions in neonatology in Denmark. Med Anthropol. 2018;37(3):253‐266. 28375646 10.1080/01459740.2017.1313841

[31] Sommer CM , Cook CM . Disrupted bonds–parental perceptions of regionalised transfer of very preterm infants: a small‐scale study. Contemp Nurse. 2015;50(2‐3):256‐266. 26514339 10.1080/10376178.2015.1114421

[32] Attar MA , Lang SW , Gates MR , Iatrow AM , Bratton SL . Back transport of neonates: effect on hospital length of stay. J Perinatol. 2005;25(11):731‐736. 16222344 10.1038/sj.jp.7211391

[33] Jung AL , Bose CL . Back transport of neonates: improved efficiency of tertiary nursery bed utilization. Pediatrics. 1983;71(6):918‐922. 6406977

[34] Richardson DK , Zupancic JA , Escobar GJ , Ogino M , Pursley DM , Mugford M . A critical review of cost reduction in neonatal intensive care II. Strategies for reduction. J Perinatol. 2001;21(2):121‐127. 11324358 10.1038/sj.jp.7200501

[35] Phibbs CS , Mortensen L . Back transporting infants from neonatal intensive care units to community hospitals for recovery care: effect on hospital charges. Pediatrics. 1992;90(1):22‐26. 1614772

[36] Fowlie PW , Booth P , Skeoch CH . Moving the preterm infant. BMJ. 2004;329(7471):904‐906. 15485974 10.1136/bmj.329.7471.904 PMC523122

[37] Gavey J . Parental perceptions of neonatal care. J Neonatal Nurs. 2007;13(5):199‐206.

[38] Donohue PK , Hussey‐Gardner B , Sulpar LJ , Fox R , Aucott SW . Parents' perception of the back‐transport of very‐low‐birth‐weight infants to community hospitals. J Perinatol. 2009;29(8):575‐581. 19262570 10.1038/jp.2009.17

[39] Hall EO . Danish parents' experiences when their new born or critically ill small child is transferred to the PICU—a qualitative study. Nurs Crit Care. 2005;10(2):90‐97. 15839240 10.1111/j.1362-1017.2005.00096.x

[40] Helder OK , Verweij JCM , van Staa A . Transition from neonatal intensive care unit to special care nurseries: experiences of parents and nurses. Pediatr Crit Care Med. 2012;13(3):305‐311. 21705956 10.1097/PCC.0b013e3182257a39

[41] Griffith AI , Smith DE , eds. Under New Public Management: Institutional Ethnographies of Changing Front‐Line Work. University of Toronto Press; 2014.

[42] Aagaard H , Hall EOC , Ludvigsen MS , Uhrenfeldt L , Fegran L . Parents' experiences of neonatal transfer. A meta‐study of qualitative research 2000–2017. Nurs Inquiry. 2018;25(3):e12231. 10.1111/nin.12231 29446189

[43] Pillay T , Modi N , Rivero‐Arias O , et al. Optimising neonatal service provision for preterm babies born between 27 and 31 weeks gestation in England (OPTI‐PREM), using national data, qualitative research and economic analysis: a study protocol. BMJ Open. 2019;9(8):029421. 10.1136/bmjopen-2019-029421 PMC6707683 31444186

[44] Bliss . What We Do. Bliss; 2020. https://www.bliss.org.uk/about-us/what-we-do

[45] Glaser BG , Strauss AL . The Discovery of Grounded Theory: Strategies Theory: Strategies for Qualitative Research. Aldine Publishing Company; 1967:292.

[46] Smith DE . The Everyday World as Problematic: A Feminist Sociology. University of Toronto Press; 1987:258.

[47] Campbell M , Gregor FM . Mapping Social Relations: A Primer in Doing Institutional Ethnography. University of Toronto Press; 2002:148.

[48] Smith DE . Institutional Ethnography as Practice. Rowman & Littlefield; 2006:280.

[49] Rankin J . Conducting analysis in institutional ethnography: guidance and cautions. Int J Qual Methods. 2017;16(1):160940691773447.

[50] Cupit C , Rankin J , Armstrong N . Taking sides with patients using institutional ethnography. J Organ Ethnogr. 2021;10(1):21‐35.

[51] Ringham C , Rankin JM , Premji SS , Marcellus L . The social organization of nurses' work with late preterm infants in non‐tertiary care settings: out of the corners of nurses' eyes. In: Premji SS , ed. Late Preterm Infants: A Guide for Nurses, Midwives, Clinicians and Allied Health Professionals. Springer International Publishing; 2019:53‐65. 10.1007/978-3-319-94352-7_5

[52] Cheah IGS . Economic assessment of neonatal intensive care. Transl Pediatr. 2019;8(3):246‐256. 31413958 10.21037/tp.2019.07.03 PMC6675687

[53] Lee SK , O'Brien K . Parents as primary caregivers in the neonatal intensive care unit. Can Med Assoc J. 2014;186(11):845‐847. 24710910 10.1503/cmaj.130818 PMC4119144

[54] Waddington C , van Veenendaal NR , O'Brien K , Patel N . Family integrated care: supporting parents as primary caregivers in the neonatal intensive care unit. Pediatr Invest. 2021;5(2):148‐154. 10.1002/ped4.12277 PMC8212757 34179713

[55] Dosani A , Murthy P , Kassam S , Rai B , Lodha AK . Parental perception of neonatal transfers from level 3 to level 2 neonatal intensive care units in Calgary, Alberta: qualitative findings. BMC Health Serv Res. 2021;21(1):981. 34535124 10.1186/s12913-021-06967-3 PMC8449487

[56] Wigert H , Berg M , Hellström AL . Parental presence when their child is in neonatal intensive care. Scand J Caring Sci. 2010;24(1):139‐146. 19508328 10.1111/j.1471-6712.2009.00697.x

[57] Woodward LJ , Bora S , Clark CAC , et al. Very preterm birth: maternal experiences of the neonatal intensive care environment. J Perinatol. 2014;34(7):555‐561. 24651730 10.1038/jp.2014.43 PMC4154363

[58] Gonya J , Harrison T , Feldman K , Stein M , Chawla N . Nursing networks in the NICU and their association with maternal stress: a pilot study. J Nurs Manag. 2019;27(2):442‐449. 30238539 10.1111/jonm.12679

[59] Baird J , Rehm RS , Hinds PS , Baggott C , Davies B . Do you know my child? Continuity of nursing care in the pediatric intensive care unit. Nurs Res. 2016;65(2):142‐150. 26938363 10.1097/NNR.0000000000000135 PMC4780357

[60] Gjengedal E , Sviland R , Moi AL , et al. Patients' quest for recognition and continuity in health care: time for a new research agenda? Scand J Caring Sci. 2019;33(4):978‐985. 31032985 10.1111/scs.12696

[61] Montori VM Turning away from industrial health care toward careful and kind care. Acad Med 2019;94(6):768‐770. 30475268 10.1097/ACM.0000000000002534

