# Supplementary material for: Parenting through place‐of‐care disruptions: A qualitative study of parents' experiences of neonatal care
Source: Health Expect. 2023 Dec 18;27(1):e13933. doi: 10.1111/hex.13933 (PMC10726285; doi:10.1111/hex.13933)
Supplement: Supplementary file 1 — Supporting information. [file HEX-27-e13933-s002.docx]

**OPTI-PREM Interview Schedule – Real time parents**

*N.B. This topic guide will be used flexibly – the interviewer will be responsive to each individual participant and will explore related issues of interest as they arise.*

*N.B. For simplicity, this topic guide refers to a singleton pregnancy but in the case of multiple birth the interviewer will ensure discussion of each baby.*

**Welcome and introduction**

- Confirm that the interview is solely for the use of the researchers and any discussions will not be communicated in any way to any care provider
- The report will pull together findings from all participants in the study and no individual will be identified.
- We like to audio-record each interview – is that ok?

**Reiterate aims of interview**

- To understand parents’ views and experiences and make sure these are included in discussions about the optimal place of care for babies born between 27 and 31 weeks of gestation.
- We are interested in your views and experiences based on the care your baby has received. If you do not feel you are able to comment on any area please say so.
- Do you have any questions before we start?

**Background to participant and baby**

- Please could you tell me a little bit about yourself?
- Prompt for: age, parity, occupation, how far away from the unit parent lives, relationship status
- Please could you tell me a little bit about your baby?
- Prompt for: gestation, single or multiple, any particular health problems

**Journey through neonatal care**

- Can you tell me the story of how your baby came to be receiving care on this neonatal unit, starting from when s/he was born?
- Prompt for any transfers in place of care that have happened
- Prompt for duration(s)

*N.B. pick up the following issues as appropriate to nature and extent of the story just told*

**Place of birth**

- How did your baby come to be born where s/he was?
- Prompt for whether mother presented at this hospital or was brought here e.g. by ambulance
- Was your baby’s place of birth as planned?
- If yes, how and by whom was it planned to be this way?
- If no, how did it come to happen as it did?
- How much, if anything did you know at that stage about neonatal units, particularly the different types/levels?

**Transfers between units**

- Can you tell me a bit more about the transfers you and your baby have experienced?
- For each transfer, prompt for:
- Type/level of unit from and to
- Geography
- Reason for transfer
- Parents’ feelings/experiences
- Have any transfers been discussed/planned that then did not happen?
- What happened?
- How was this for you?
- Do you think any more transfers might happen for you baby?
- Why/why not?
- How do you feel about that?

**Parents’ involvement in decision-making**

- Have you been involved in any decisions about whether to move your baby?
- If yes:
- Tell me about how that happened
- What kinds of things were important to you in that decision?
- If no:
- Tell me about how that happened?
- Would you like to have been more involved?

**Relationship with healthcare professionals**

- Tell me about your relationship with the healthcare professionals that have been looking after your baby, particularly in relation to any possible or actual transfers.
- Prompt about transfers out: being informed/involved
- Prompt about transfers in: settling in to new unit and any changes

**Impact on parents**

- Tell me about some of the ways this is impacting on you, particularly in relation to the place of care for your baby.
- Prompt for: financial, logistics, work, family/other children, relationship with partner

**Improving parents’ experiences**

- Thinking particularly about decisions about and any changes in your baby’s place of care, what has worked well for you?
- Prompt for more detail on why/how
- What could have been better?
- Why was this a problem?
- How could it be improved?

**Anything not covered?**

- Is there anything that we haven’t covered in the interview that you think we should know or think about for this project?

**Closing and thanks**

- Check that the participant is still happy for us to use all the information provided and offer the possibility to erase sections of the recording.
- Thank for their time and contribution.
