# Supplementary material for: Parenting through place‐of‐care disruptions: A qualitative study of parents' experiences of neonatal care
Source: Health Expect. 2023 Dec 18;27(1):e13933. doi: 10.1111/hex.13933 (PMC10726285; doi:10.1111/hex.13933)
Supplement: Supplementary file 2 — Supporting information. [file HEX-27-e13933-s001.docx]

**OPTI-PREM Interview Schedule – Retrospective parents**

*N.B. This topic guide will be used flexibly – the interviewer will be responsive to each individual participant and will explore related issues of interest as they arise.*

*N.B. For simplicity, this topic guide refers to a singleton pregnancy but in the case of multiple birth the interviewer will ensure discussion of each baby.*

**Welcome and introduction**

- Confirm that the interview is solely for the use of the researchers and any discussions will not be communicated in any way to any care provider
- The report will pull together findings from all participants in the study and no individual will be identified.
- We like to audio-record each interview – is that ok?

**Reiterate aims of interview**

- To understand parents’ views and experiences and make sure these are included in discussions about the optimal place of care for babies born between 27 and 31 weeks of gestation.
- We are interested in your views and experiences based on the care your baby received. If you do not feel you are able to comment on any area please say so.
- Do you have any questions before we start?

**Background to participant and baby**

- Please could you tell me a little bit about yourself?
- Prompt for: age, parity, occupation, relationship status (any changes between now and time baby was receiving neonatal care?)
- Please could you tell me a little bit about your baby?
- Prompt for: gestation, single or multiple, any particular health problems

**Journey through neonatal care**

- Can you tell me the story of your baby’s neonatal care, starting from when s/he was born?
- Prompt for any transfers in place of care that happened
- Prompt for duration(s)

*N.B. pick up the following issues as appropriate to nature and extent of the story just told*

**Place of birth**

- How did your baby come to be born where s/he was?
- Prompt for whether mother presented at this hospital or was brought there e.g. by ambulance
- Was your baby’s place of birth as planned?
- If yes, how and by whom was it planned to be this way?
- If no, how did it come to happen as it did?
- How much, if anything did you know at that stage about neonatal units, particularly the different types/levels?

**Transfers between units**

- Can you tell me a bit more about the transfers you and your baby experienced?
- For each transfer, prompt for:
- Type/level of unit from and to
- Geography
- Reason for transfer
- Parents’ feelings/experiences
- Were any transfers discussed/planned that then did not happen?
- What happened?
- How was this for you?

**Parents’ involvement in decision-making**

- Were you involved in any decisions about whether to move your baby?
- If yes:
- Tell me about how that happened
- What kinds of things were important to you in that decision?
- If no:
- Tell me about how that happened?
- Would you like to have been more involved?

**Relationship with healthcare professionals**

- Tell me about your relationship with the healthcare professionals that looked after your baby, particularly in relation to any possible or actual transfers.
- Prompt about transfers out: being informed/involved
- Prompt about transfers in: settling in to new unit and any changes

**Impact on parents**

- Tell me about some of the ways this all impacted on you, particularly in relation to the place of care for your baby.
- Prompt for: financial, logistics, work, family/other children, relationship with partner

**Improving parents’ experiences**

- Thinking particularly about decisions about and any changes in your baby’s place of care, what worked well for you?
- Prompt for more detail on why/how
- What could have been better?
- Why was this a problem?
- How could it be improved?
- Now that a bit of time has passed, do you think about anything differently now that you’re not right in the middle of it?
- Why do you think that is?

**Anything not covered?**

- Is there anything that we haven’t covered in the interview that you think we should know or think about for this project?

**Closing and thanks**

- Check that the participant is still happy for us to use all the information provided and offer the possibility to erase sections of the recording.
- Thank for their time and contribution.
